# Supplementary material for: Environmental perturbations lead to extensive directional shifts in RNA processing
Source: PLoS Genet. 2017 Oct 12;13(10):e1006995. doi: 10.1371/journal.pgen.1006995 (PMC5667937; doi:10.1371/journal.pgen.1006995)
Supplement: S1 Fig — This is a figure depicting the colors that are used to denote each of the 32 treatments throughout the manuscript. (PDF) [file pgen.1006995.s001.pdf]

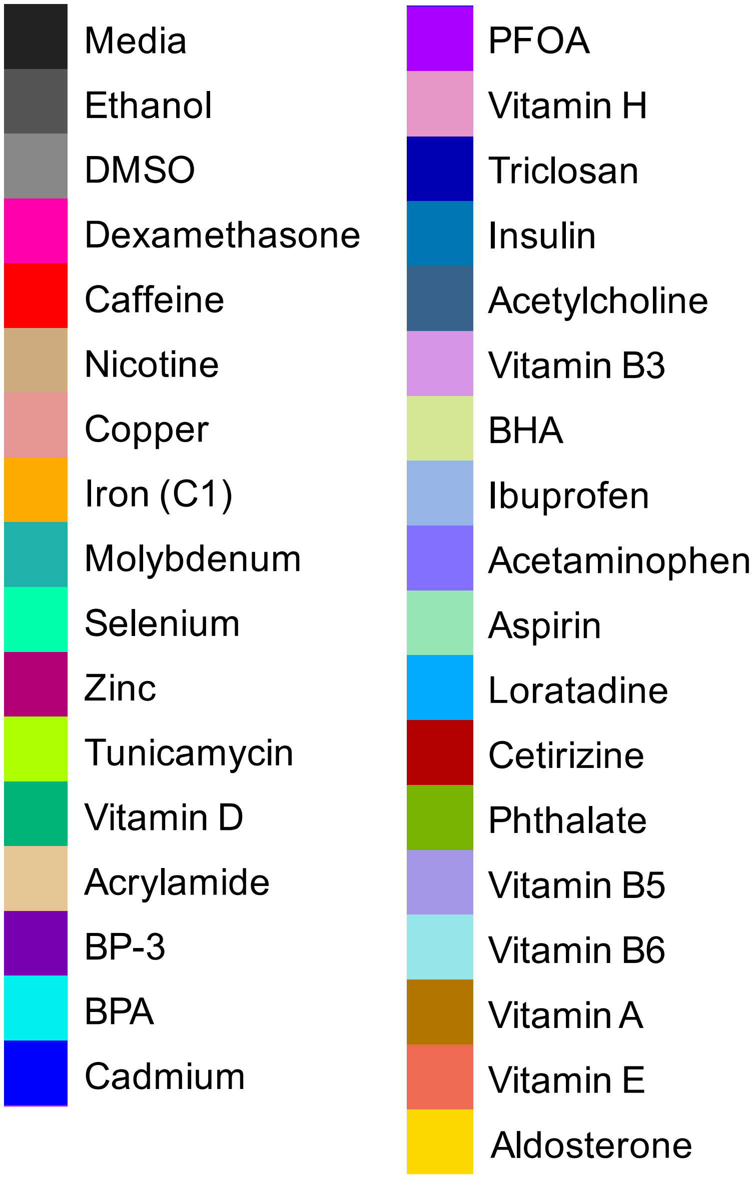

S1 Fig. **Color legend for treatments.**

This is a figure depicting the colors that are used to denote each of the 32 treatments throughout the manuscript.
