# Supplementary material for: Environmental perturbations lead to extensive directional shifts in RNA processing
Source: PLoS Genet. 2017 Oct 12;13(10):e1006995. doi: 10.1371/journal.pgen.1006995 (PMC5667937; doi:10.1371/journal.pgen.1006995)
Supplement: S2 Fig — This is a two panel figure depicting the following: A) This plot indicates the read coverage for each library assessed for change in RNA processing events. The y-axis is the number of significant shifts for each library. Each point is a library and the color indicates the treatment for each sample. The y-axis is the number of significant shifts to which that particular library contributes a Ψ value to the calculation. Because significant shifts are calculated across individuals, libraries derived from the same cell type and treatment but in different individuals will have similar number of RNA processing shifts. Spearman’s correlation and significance is indicated. B) Each point depicts the number of differentially expressed genes (x-axis) and the number of significant RNA processing event changes (y-axis). In this plot, each point indicates values for a cell type and treatment because differential expression and RNA processing shifts are both calculated across three individuals. Each point is colored by the treatment. (PDF) [file pgen.1006995.s002.pdf]

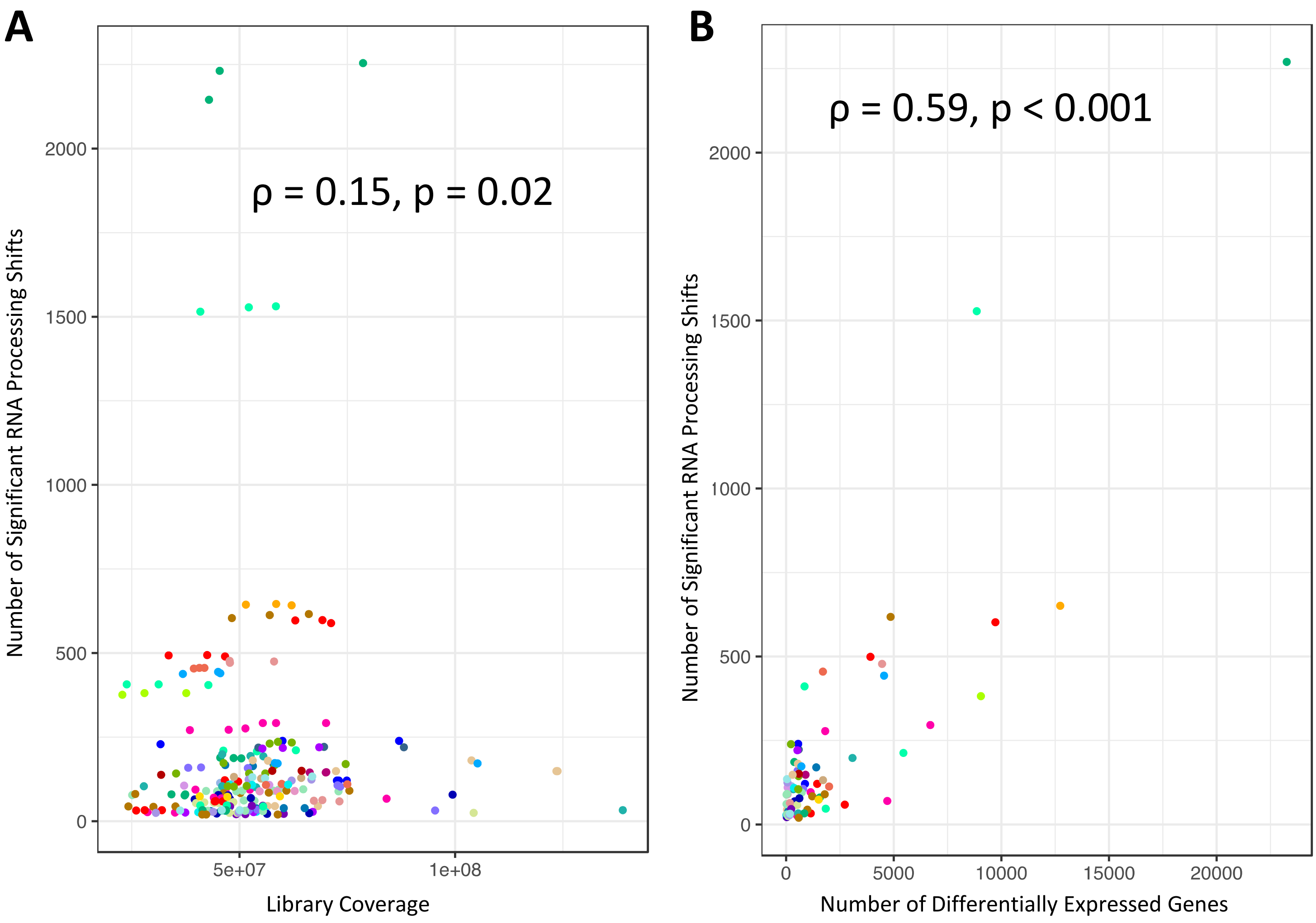

S2 Fig. **Correlation of the Number of Significant Shifts in Events with Sequencing Coverage and Number of DEG.**

This is a two panel figure depicting the following: A) This plot indicates the read coverage for each library assessed for change in RNA processing events. The y-axis is the number of significant shifts for each library. Each point is a library and the color indicates the treatment for each sample. The y-axis is the number of significant shifts to which that particular library contributes a Ψ value to the calculation. Because significant shifts are calculated across individuals, libraries derived from the same cell type and treatment but in different individuals will have similar number of RNA processing shifts. Spearman's correlation and significance is indicated. B) Each point depicts the number of differentially expressed genes (x-axis) and the number of significant RNA processing event changes (y-axis). In this plot, each point indicates values for a cell type and treatment because differential expression and RNA processing shifts are both calculated across three individuals. Each point is colored by the treatment.
