# Supplementary material for: Environmental perturbations lead to extensive directional shifts in RNA processing
Source: PLoS Genet. 2017 Oct 12;13(10):e1006995. doi: 10.1371/journal.pgen.1006995 (PMC5667937; doi:10.1371/journal.pgen.1006995)
Supplement: S3 Fig — These graphs show the correlation of gene expression changes and significant RNA processing shifts (using ΔΨ) following various treatments. Each point represents one RNA processing shift in a given cell type, with treatment indicated above the graph. Specifically, A is a graph for genes with shifts in ALE usage following iron, B) shows genes with shifts in ALE following selenium, C) shows genes with shifts in SE following vitamin D exposure and D) shows genes with shifts in AFE usage following treatment with copper. (PDF) [file pgen.1006995.s003.pdf]

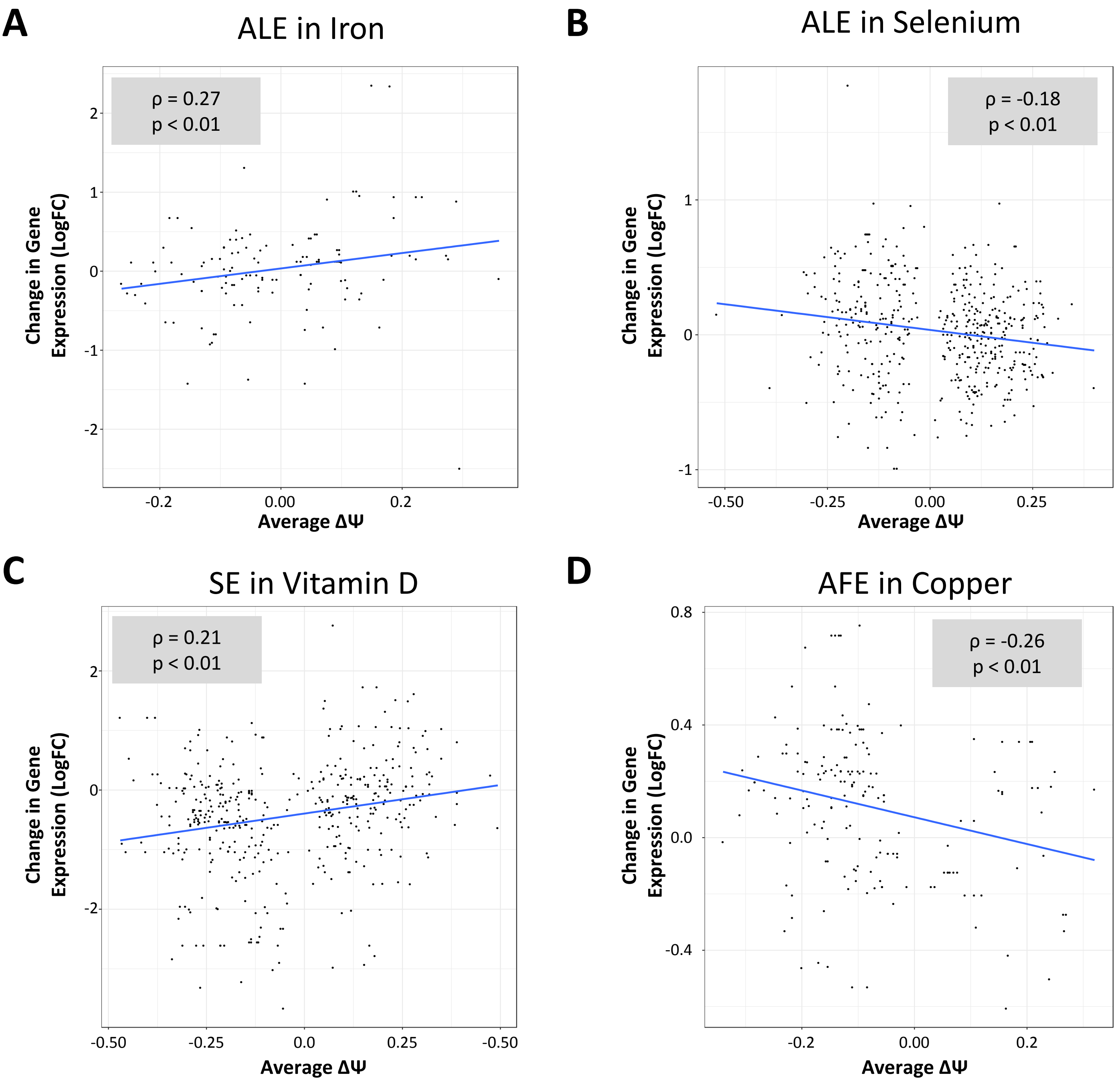

S3 Fig. **Examples of Correlation between Differential Gene Expression and Differential RNA Processing.**

These graphs show the correlation of gene expression changes and significant RNA processing shifts (using $\Delta\Psi$) following various treatments. Each point represents one RNA processing shift in a given cell type, with treatment indicated above the graph. Specifically, A is a graph for genes with shifts in ALE usage following iron, B) shows genes with shifts in ALE following selenium, C) shows genes with shifts in SE following vitamin D exposure and D) shows genes with shifts in AFE usage following treatment with copper.
