# Supplementary material for: Environmental perturbations lead to extensive directional shifts in RNA processing
Source: PLoS Genet. 2017 Oct 12;13(10):e1006995. doi: 10.1371/journal.pgen.1006995 (PMC5667937; doi:10.1371/journal.pgen.1006995)
Supplement: S4 Fig — A) This plot indicates the direction of shifts for RI in the different cell types following exposure to vitamin D. The height of each bar shows the proportion of significant event shifts. A binomial test compares the proportion of significant events to the average across all environments. Each bar is then broken in two with the shaded region showing the proportion of the significant changes that shifted towards a positive ΔΨ (inclusion of intron) while the white region of each bar is the proportion of sites with a negative ΔΨ. If there is a significant departure from the expected 50:50 for positive to negative ΔΨ (tested using a binomial test), the row of boxes shows this (red denotes enrichment for positive ΔΨ, blue for negative ΔΨ). B) These plots correspond to those in A and show the ECDF of the ΔΨ across all event shifts, not just the significant event shifts. C) This plot is similar to A) but it shows the direction of shifts for AFE in various cell types responding to treatment with insulin (with positive ΔΨ equating to inclusion of the upstream AFE). D) Corresponding ECDF plots to C) that show the ΔΨ across all event shifts. (PDF) [file pgen.1006995.s004.pdf]

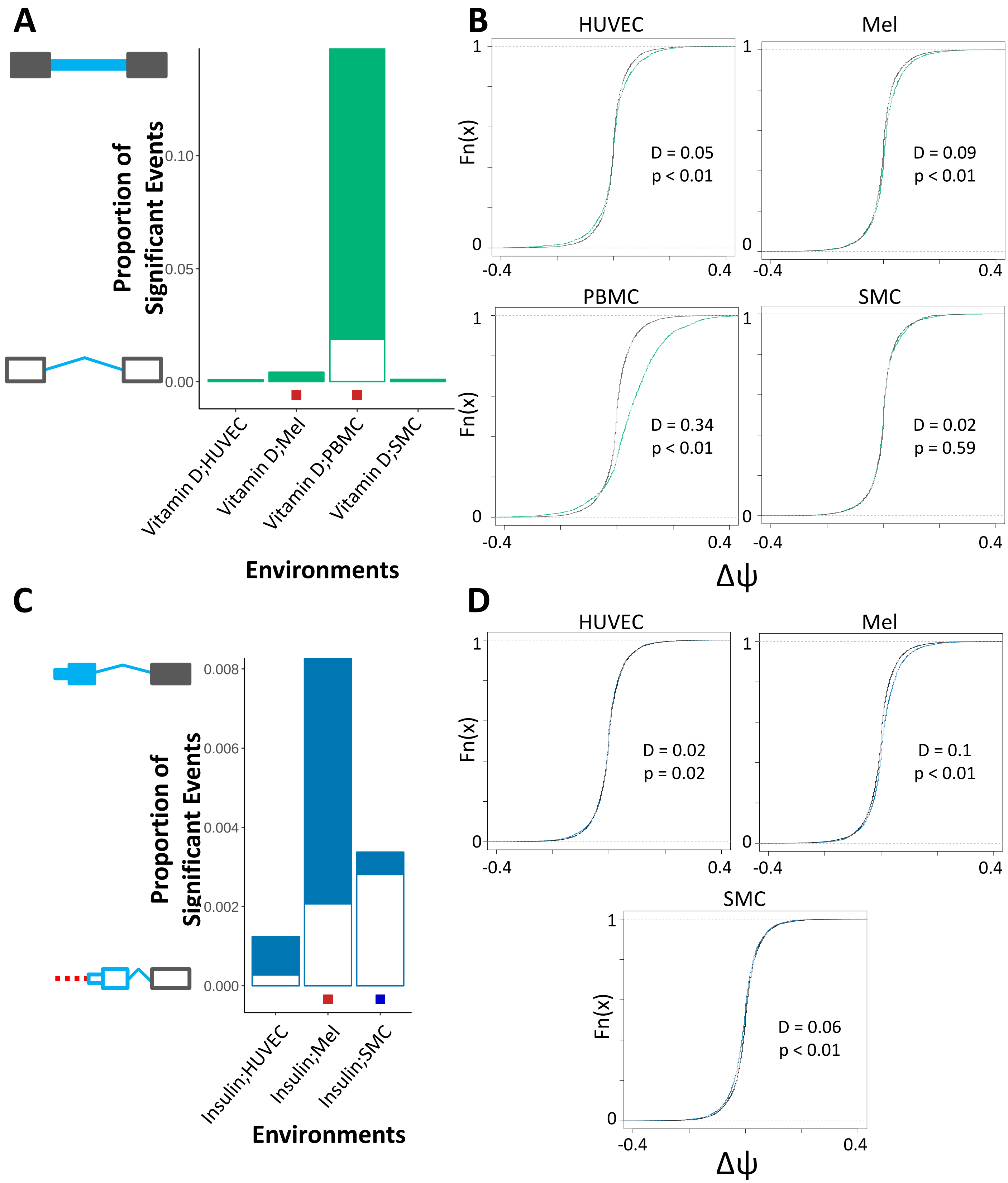

S4 Fig. **Examples of Different and Shared Directional Changes in RI Following Treatments.**

A) This plot indicates the direction of shifts for RI in the different cell types following exposure to vitamin D. The height of each bar shows the proportion of significant event shifts. A binomial test compares the proportion of significant events to the average across all environments. Each bar is then broken in two with the shaded region showing the proportion of the significant changes that shifted towards a positive $\Delta\Psi$ (inclusion of intron) while the white region of each bar is the proportion of sites with a negative $\Delta\Psi$. If there is a significant departure from the expected 50:50 for positive to negative $\Delta\Psi$ (tested using a binomial test), the row of boxes shows this (red denotes enrichment for positive $\Delta\Psi$, blue for negative $\Delta\Psi$). B) These plots correspond to those in A and show the ECDF of the $\Delta\Psi$ across all event shifts, not just the significant event shifts. C) This plot is similar to A) but it shows the direction of shifts for AFE in various cell types responding to treatment with insulin (with positive $\Delta\Psi$ equating to inclusion of the upstream AFE). D) Corresponding ECDF plots to C) that show the $\Delta\Psi$ across all event shifts.
