# Supplementary material for: Environmental perturbations lead to extensive directional shifts in RNA processing
Source: PLoS Genet. 2017 Oct 12;13(10):e1006995. doi: 10.1371/journal.pgen.1006995 (PMC5667937; doi:10.1371/journal.pgen.1006995)
Supplement: S5 Fig — These plots indicate the direction of shift for 2 event types: ALE (left) and TandemUTR (Right). Each plot shows environments for which these events were tested and more than 10 significant events were identified. The height of each bar shows the proportion of significant event shifts for each environment while the dotted line indicates the average proportion of significant events across all environments shown here. Each bar is then broken in two with the shaded region showing the proportion of the significant changes that shifted towards a positive ΔΨ (inclusion longer UTR or downstream ALE) while the white region of each bar is the proportion of sites with a negative ΔΨ. The column of boxes shows if there is a departure from the expected 50:50 for positive to negative ΔΨ (tested using a binomial test). Red denotes enrichment for ΔΨ > 0 and blue for ΔΨ < 0). (PDF) [file pgen.1006995.s005.pdf]

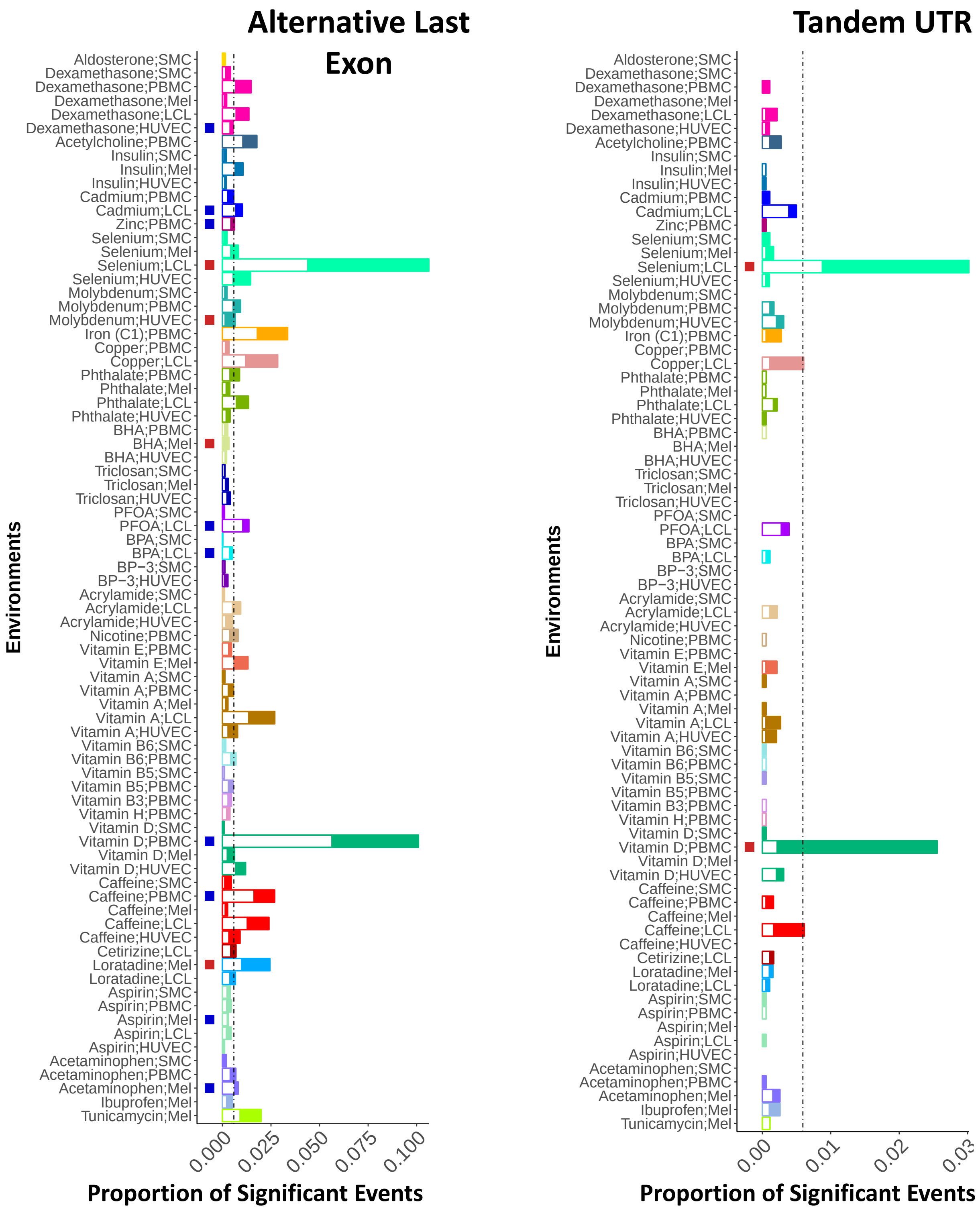

S5 Fig. **Additional Direction of Shift in Events Following Treatment.**

These plots indicate the direction of shift for 2 event types: ALE (left) and TandemUTR (Right). Each plot shows environments for which these events were tested and more than 10 significant events were identified. The height of each bar shows the proportion of significant event shifts for each environment while the dotted line indicates the average proportion of significant events across all environments shown here. Each bar is then broken in two with the shaded region showing the proportion of the significant changes that shifted towards a positive $\Delta\Psi$ (inclusion longer UTR or downstream ALE) while the white region of each bar is the proportion of sites with a negative $\Delta\Psi$. The column of boxes shows if there is a departure from the expected 50:50 for positive to negative $\Delta\Psi$ (tested using a binomial test). Red denotes enrichment for $\Delta\Psi > 0$ and blue for $\Delta\Psi < 0$).
