# Supplementary material for: Environmental perturbations lead to extensive directional shifts in RNA processing
Source: PLoS Genet. 2017 Oct 12;13(10):e1006995. doi: 10.1371/journal.pgen.1006995 (PMC5667937; doi:10.1371/journal.pgen.1006995)
Supplement: S1 Table — Cellular environments are defined by combinations of 5 cell types, 32 treatments and 3 controls. (PDF) [file pgen.1006995.s006.pdf]

Treatment Code	Full Treatment Name	Treatment Name	HUVEC	LCL	Mel	PBMC	SMC
CO1	Media (Water)	CO1	X	X	X	X	X
CO2	Ethanol	CO2	X	X	X	X	X
CO3	DMSO	CO3			X		
T2C1	Biotin	Vitamin H				X	
T3C1	Niacin (Nicotinic acid)	Vitamin B3				X	
T4C1	Panthothenic acid	Vitamin B5				X	X
T5C1	Vitamin B6	Vitamin B6				X	X
T6C1	Retinoic Acid	Vitamin A	X	X	X	X	X
T7C1	Vitamin E	Vitamin E			X	X	
T9C1	Aldosterone	Aldosterone					X
T12C1	Dexamethasone	Dexamethasone	X	X	X	X	X
T13C1	Caffeine	Caffeine	X	X	X	X	X
T14C1	Nicotine	Nicotine				X	
T15C1	Copper (Copper (II) Chloride)	Copper		X		X	
T16C1	Iron (Iron (III) Chloride)(5mM)	Iron (C1)				X	
T18C1	Molybdenum (Molybdenum (V) Chloride)	Molybdenum	X			X	X
T19C1	Selenium (Sodium Selenite)	Selenium	X	X	X		X
T20C1	Zinc (Zinc Chloride)	Zinc				X	
T21C1	Tunicamycin	Tunicamycin			X		
T23C1	Vitamin D	Vitamin D	X		X	X	X
T24C1	Acrylamide	Acrylamide	X	X			X
T25C1	Benzophenone-3 (BP-3)	BP-3	X				X
T26C1	Bisphenol A (BPA)	BPA		X			X
T27C1	Cadmium	Cadmium		X		X	
T29C1	Perfluorooctanoic Acid (Nonstick Cookware)	PFOA		X			X
T30C1	Triclosan (Disinfectant)	Triclosan	X		X		X
T33C1	Insulin	Insulin	X		X		X
T37C1	Acetylcholine	Acetylcholine				X	
T40C1	Butylated HydroxyAnisole	BHA	X		X	X	
T41C1	Ibuprofen (NSAID)	Ibuprofen			X		
T42C1	Acetaminophen (NSAID)	Acetaminophen			X	X	X
T43C1	Aspirin (NSAID)	Aspirin	X	X	X	X	X
T44C1	Loratadine (Allergy)	Loratadine		X	X		
T45C1	Cetirizine (Allergy)	Cetirizine		X			
T47C1	Mono-n-butyl Phthalate	Phthalate	X	X	X	X	

S1 Table. **List of 89 Environments Analyzed for RNA Processing Events.**

Cellular environments are defined by combinations of 5 cell types, 32 treatments and 3 controls.
