# Supplementary material for: Environmental perturbations lead to extensive directional shifts in RNA processing
Source: PLoS Genet. 2017 Oct 12;13(10):e1006995. doi: 10.1371/journal.pgen.1006995 (PMC5667937; doi:10.1371/journal.pgen.1006995)
Supplement: S3 Table — Genes that contained events that shifted in at least one environment were compared to all genes that were tested for an RNA processing event shift. Analysis was performed using GeneTrail [41]. (PDF) [file pgen.1006995.s008.pdf]

Subcategory	Alternative Subcategory Name	Expected	Observed	p-value (FDR)	Enrichment
intracellular	GO:0005622	2288.25	2442	9.60E-12	up
nucleus	GO:0005634	1082.89	1244	1.26E-10	up
intracellular part	GO:0044424	2224.95	2373	1.40E-10	up
organelle	GO:0043226	1905.38	2061	5.47E-10	up
intracellular organelle	GO:0043229	1902.97	2059	5.47E-10	up
RNA binding	GO:0003723	175.093	246	1.20E-08	up
protein binding	GO:0005515	1686.26	1834	1.33E-08	up
nucleobase, nucleoside, nucleotide and nucleic acid metabolic process	GO:0006139	875.12	1006	4.37E-08	up
membrane-bounded organelle	GO:0043227	1738.54	1877	1.39E-07	up
intracellular membrane-bounded organelle	GO:0043231	1737.17	1875	1.53E-07	up
organelle organization	GO:0006996	339.866	428	1.63E-07	up
cellular metabolic process	GO:0044237	1518.39	1657	1.63E-07	up
cellular nitrogen compound metabolic process	GO:0034641	929.127	1054	3.35E-07	up
cellular macromolecule metabolic process	GO:0044260	1191.59	1324	3.35E-07	up
cellular process	GO:0009987	2252.47	2371	3.88E-07	up
gene expression	GO:0010467	764.01	880	4.85E-07	up
cellular component organization	GO:0016043	603.709	706	2.06E-06	up
nitrogen compound metabolic process	GO:0006807	947.703	1064	3.35E-06	up
intracellular organelle part	GO:0044446	1079.8	1200	3.35E-06	up
organelle part	GO:0044422	1092.18	1212	3.72E-06	up
nucleic acid binding	GO:0003676	624.693	723	8.76E-06	up
binding	GO:0005488	2363.58	2465	9.86E-06	up
nucleic acid metabolic process	GO:0090304	761.946	863	2.62E-05	up
regulation of cellular metabolic process	GO:0031323	715.163	812	4.49E-05	up
regulation of primary metabolic process	GO:0080090	680.764	775	5.46E-05	up
regulation of metabolic process	GO:0019222	753.002	850	6.06E-05	up
nucleotide binding	GO:0000166	497.071	580	6.66E-05	up
nuclear part	GO:0044428	473.335	554	7.94E-05	up
cytoplasm	GO:0005737	1629.84	1740	8.22E-05	up
regulation of macromolecule metabolic process	GO:0060255	661.5	752	9.97E-05	up
macromolecule metabolic process	GO:0043170	1283.1	1390	0.000125	up
non-membrane-bounded organelle	GO:0043228	565.87	650	0.000134	up
intracellular non-membrane-bounded organelle	GO:0043232	565.87	650	0.000134	up
primary metabolic process	GO:0044238	1537.65	1645	0.000141	up
cellular biosynthetic process	GO:0044249	851.041	946	0.000198	up

Subcategory	Alternative Subcategory Name	Expected	Observed	p-value (FDR)	Enrichment
cellular macromolecule biosynthetic process	GO:0034645	697.619	784	0.000377	up
regulation of cellular protein metabolic process	GO:0032268	132.438	175	0.000379	up
biosynthetic process	GO:0009058	869.616	961	0.000478	up
cellular protein metabolic process	GO:0044267	563.806	642	0.000535	up
posttranscriptional regulation of gene expression	GO:0010608	55.383	83	0.000547	up
metabolic process	GO:0008152	1674.22	1773	0.000639	up
histone deacetylation	GO:0016575	5.84789	15	0.000751	up
chromatin organization	GO:0006325	90.8144	125	0.000767	up
macromolecule biosynthetic process	GO:0009059	710.347	793	0.000846	up
regulation of gene expression	GO:0010468	560.71	636	0.000961	up
transcription factor binding	GO:0008134	123.494	162	0.001161	up
translation	GO:0006412	97.0062	131	0.001395	up
chromosome organization	GO:0051276	122.118	160	0.001395	up
response to stress	GO:0006950	364.977	426	0.001729	up
regulation of protein metabolic process	GO:0051246	149.981	191	0.001729	up
regulation of protein ubiquitination	GO:0031396	33.7114	54	0.001759	up
regulation of organic acid transport	GO:0032890	3.43994	10	0.002049	up
cell death	GO:0008219	270.723	323	0.002405	up
negative regulation of metabolic process	GO:0009892	184.037	228	0.002405	up
death	GO:0016265	270.723	323	0.002405	up
chromatin modification	GO:0016568	81.8705	112	0.002433	up
cell cycle	GO:0007049	238.732	288	0.002436	up
negative regulation of macromolecule metabolic process	GO:0010605	173.373	216	0.002436	up
regulation of nitrogen compound metabolic process	GO:0051171	568.278	639	0.002482	up
regulation of cellular process	GO:0050794	1204.67	1293	0.002509	up
negative regulation of protein ubiquitination	GO:0031397	22.7036	39	0.002558	up
regulation of nucleobase, nucleoside, nucleotide and nucleic acid metabolic process	GO:0019219	563.806	633	0.003159	up
regulation of biological process	GO:0050789	1263.49	1351	0.003159	up
protein amino acid deacetylation	GO:0006476	7.56786	17	0.003473	up
nuclear lumen	GO:0031981	371.169	429	0.003671	up
transcription regulator activity	GO:0030528	281.731	333	0.003717	up
regulation of macromolecule biosynthetic process	GO:0010556	543.854	611	0.003888	up
cytosol	GO:0005829	315.442	369	0.003956	up

Subcategory	Alternative Subcategory Name	Expected	Observed	p-value (FDR)	Enrichment
heterogeneous nuclear ribonucleoprotein complex	GO:0030530	4.12793	11	0.004376	up
molecular_function	GO:0003674	2764.68	2817	0.00461	up
chromosome	GO:0005694	112.83	146	0.00461	up
translational elongation	GO:0006414	24.7676	41	0.00461	up
lysine N-methyltransferase activity	GO:0016278	11.0078	22	0.00473	up
protein-lysine N-methyltransferase activity	GO:0016279	11.0078	22	0.00473	up
histone-lysine N-methyltransferase activity	GO:0018024	11.0078	22	0.00473	up
regulation of biosynthetic process	GO:0009889	563.462	630	0.004935	up
RNA processing	GO:0006396	145.165	182	0.005224	up
regulation of cellular biosynthetic process	GO:0031326	560.022	626	0.005288	up
biological regulation	GO:0065007	1328.16	1411	0.006572	up
regulation of translation	GO:0006417	33.0234	51	0.006864	up
cell cycle process	GO:0022402	160.301	198	0.006872	up
transcription cofactor activity	GO:0003712	84.9665	113	0.006881	up
programmed cell death	GO:0012501	249.051	295	0.006889	up
mRNA binding	GO:0003729	14.7917	27	0.007265	up
double-stranded RNA binding	GO:0003725	8.59984	18	0.007548	up
apoptosis	GO:0006915	246.644	292	0.007548	up
histone methyltransferase activity	GO:0042054	12.7278	24	0.00798	up
positive regulation of cellular process	GO:0048522	417.952	474	0.009184	up
nucleobase, nucleoside and nucleotide metabolic process	GO:0055086	113.862	145	0.009987	up
protein methyltransferase activity	GO:0008276	16.5117	29	0.010365	up
negative regulation of cellular process	GO:0048523	378.049	431	0.010921	up
covalent chromatin modification	GO:0016569	45.0632	65	0.011098	up
negative regulation of cellular metabolic process	GO:0031324	168.213	205	0.011541	up
regulation of cell cycle	GO:0051726	106.294	136	0.011616	up
histone modification	GO:0016570	44.3752	64	0.012068	up
biological_process	GO:0008150	2579.95	2638	0.012605	up
cellular response to stimulus	GO:0051716	235.636	278	0.012917	up
response to protein stimulus	GO:0051789	25.1115	40	0.012917	up
cytoskeleton organization	GO:0007010	117.302	148	0.013022	up
proteolysis involved in cellular protein catabolic process	GO:0051603	85.6544	112	0.013917	up
nucleoside phosphate metabolic process	GO:0006753	108.702	138	0.014617	up
nucleotide metabolic process	GO:0009117	108.702	138	0.014617	up
protein metabolic process	GO:0019538	638.108	701	0.015009	up

Subcategory	Alternative Subcategory Name	Expected	Observed	p-value (FDR)	Enrichment
protein metabolic process	GO:0019538	638.108	701	0.015009	up
regulation of cellular catabolic process	GO:0031329	52.2871	73	0.015009	up
cellular response to stress	GO:0033554	166.493	202	0.015062	up
negative regulation of biological process	GO:0048519	407.289	460	0.015062	up
positive regulation of biological process	GO:0048518	453.04	508	0.015159	up
ubiquitin-dependent protein catabolic process	GO:0006511	74.6466	99	0.015353	up
membrane organization	GO:0061024	95.6303	123	0.015353	up
cellular protein catabolic process	GO:0044257	85.9984	112	0.015415	up
transcription activator activity	GO:0016563	96.6622	124	0.016192	up
proteasomal ubiquitin-dependent protein catabolic process	GO:0043161	43.3432	62	0.016858	up
methyltransferase complex	GO:0034708	14.7917	26	0.01719	up
histone methyltransferase complex	GO:0035097	14.7917	26	0.01719	up
chromatin remodeling	GO:0006338	14.1037	25	0.018331	up
cellular membrane organization	GO:0016044	95.2863	122	0.01881	up
proteasomal protein catabolic process	GO:0010498	44.3752	63	0.018917	up
mRNA processing	GO:0006397	80.4945	105	0.020165	up
transcription	GO:0006350	518.055	574	0.020779	up
ribonucleotide binding	GO:0032553	407.289	458	0.020779	up
purine ribonucleotide binding	GO:0032555	407.289	458	0.020779	up
cytoskeletal part	GO:0044430	187.821	224	0.020779	up
response to biotic stimulus	GO:0009607	78.0866	102	0.021864	up
purine nucleotide binding	GO:0017076	423.8	475	0.021992	up
chromosomal part	GO:0044427	92.1903	118	0.021992	up
RNA metabolic process	GO:0016070	516.679	572	0.02208	up
transcription repressor activity	GO:0016564	71.2067	94	0.02208	up
ribonucleoprotein complex	GO:0030529	123.494	153	0.02208	up
cytoplasmic part	GO:0044444	1116.26	1187	0.02208	up
modification-dependent protein catabolic process	GO:0019941	75.6786	99	0.022912	up
protein catabolic process	GO:0030163	97.6942	124	0.022912	up
modification-dependent macromolecule catabolic process	GO:0043632	75.6786	99	0.022912	up
protein modification by small protein conjugation or removal	GO:0070647	91.5023	117	0.022912	up
negative regulation of ligase activity	GO:0051352	18.9197	31	0.023935	up
negative regulation of ubiquitin-protein ligase activity	GO:0051444	18.9197	31	0.023935	up
organelle lumen	GO:0043233	452.352	504	0.024747	up

Subcategory	Alternative Subcategory Name	Expected	Observed	p-value (FDR)	Enrichment
histone deacetylase activity	GO:0004407	4.12793	10	0.02505	up
protein deacetylase activity	GO:0033558	4.12793	10	0.02505	up
response to unfolded protein	GO:0006986	14.4477	25	0.02556	up
nucleoplasm	GO:0005654	237.356	276	0.027426	up
regulation of transcription	GO:0045449	493.287	546	0.027426	up
negative regulation of ubiquitin-protein ligase activity involved in mitotic cell cycle	GO:0051436	17.5437	29	0.027426	up
regulation of catabolic process	GO:0009894	60.5429	81	0.027878	up
regulation of intracellular protein kinase cascade	GO:0010627	73.6147	96	0.028092	up
protein ubiquitination	GO:0016567	73.6147	96	0.028092	up
M phase of mitotic cell cycle	GO:0000087	71.8947	94	0.028269	up
macromolecular complex	GO:0032991	672.164	731	0.028269	up
N-methyltransferase activity	GO:0008170	16.8557	28	0.02864	up
organelle fission	GO:0048285	72.9267	95	0.030187	up
macromolecule modification	GO:0043412	425.176	474	0.030826	up
mRNA metabolic process	GO:0016071	95.9743	121	0.030859	up
Golgi apparatus	GO:0005794	191.605	226	0.031185	up
post-translational protein modification	GO:0043687	351.218	396	0.032351	up
positive regulation of cellular metabolic process	GO:0031325	199.172	234	0.032496	up
nuclear division	GO:0000280	70.5187	92	0.032634	up
mitosis	GO:0007067	70.5187	92	0.032634	up
RNA splicing	GO:0008380	76.7106	99	0.033302	up
nucleoside-triphosphatase regulator activity	GO:0060589	106.982	133	0.033302	up
intracellular organelle lumen	GO:0070013	445.816	495	0.033302	up
polysome	GO:0005844	3.09594	8	0.036019	up
regulation of ligase activity	GO:0051340	23.3916	36	0.036302	up
mitotic cell cycle	GO:0000278	123.494	151	0.036472	up
membrane-enclosed lumen	GO:0031974	458.888	508	0.036817	up
ATP binding	GO:0005524	338.834	382	0.037558	up
melanosome	GO:0042470	27.5195	41	0.038419	up
pigment granule	GO:0048770	27.5195	41	0.038419	up
regulation of molecular function	GO:0065009	223.252	259	0.038419	up
M phase	GO:0000279	95.9743	120	0.040551	up
GTPase regulator activity	GO:0030695	104.918	130	0.040551	up
regulation of catalytic activity	GO:0050790	185.413	218	0.040551	up

Subcategory	Alternative Subcategory Name	Expected	Observed	p-value (FDR)	Enrichment
response to stimulus	GO:0050896	606.117	660	0.040551	up
adenyl ribonucleotide binding	GO:0032559	343.306	386	0.041464	up
microtubule organizing center	GO:0005815	71.2067	92	0.041957	up
muscle tissue development	GO:0060537	32.6794	47	0.04449	up
striated muscle tissue development	GO:0014706	30.2715	44	0.046665	up
adenyl nucleotide binding	GO:0030554	359.129	402	0.046665	up
nuclear speck	GO:0016607	31.9914	46	0.048643	up

S3 Table. **Gene Ontology of Events that Significantly Shift Following Environmental Perturbation.**

Genes that contained events that shifted in at least one environment were compared to all genes that were tested for an RNA processing event shift. Analysis was performed using GeneTrail [41].
