# Supplementary material for: Environmental perturbations lead to extensive directional shifts in RNA processing
Source: PLoS Genet. 2017 Oct 12;13(10):e1006995. doi: 10.1371/journal.pgen.1006995 (PMC5667937; doi:10.1371/journal.pgen.1006995)
Supplement: S4 Table — Spearman’s correlations were calculated between log-fold change in gene expression and ΔΨ for genes containing significant shifts in RNA processing. Only treatments with p-value < 0.05 are reported here. (PDF) [file pgen.1006995.s009.pdf]

Treatment	Spearman’s ρ	p-value	Type
Vitamin D	0.146	0.0069	RI
Loratadine	0.2513	0.0308	RI
Aspirin	-0.1883	0.0421	AFE
Copper	-0.2606	5.00E-04	AFE
Caffeine	-0.22	3.00E-04	AFE
Selenium	-0.0861	0.0336	AFE
Cadmium	-0.206	0.0187	AFE
Iron	-0.3228	1.00E-04	AFE
Vitamin B3	-0.4707	0.0029	AFE
Vitamin H	-0.3888	0.0144	AFE
Selenium	-0.1817	1.00E-04	ALE
Loratadine	0.1781	0.0469	ALE
Iron	0.2711	0.0022	ALE
Vitamin D	0.2114	< 0.001	SE
Selenium	-0.3211	0.0149	TandemUTR

S4 Table. **Table of Correlation between Differential Gene Expression and Differential RNA Processing.**

Spearman's correlations were calculated between log-fold change in gene expression and $\Delta\Psi$ for genes containing significant shifts in RNA processing. Only treatments with *p*-value < 0.05 are reported here.
