# Supplementary material for: Environmental perturbations lead to extensive directional shifts in RNA processing
Source: PLoS Genet. 2017 Oct 12;13(10):e1006995. doi: 10.1371/journal.pgen.1006995 (PMC5667937; doi:10.1371/journal.pgen.1006995)
Supplement: S5 Table — Log-fold changes in gene expression of 14 splicing factors is correlated to average ΔΨ across three individuals for RI events. (PDF) [file pgen.1006995.s010.pdf]

Splicing Factor	Gene Symbol	BH FDR	Spearman's ρ	Splice Type
ENSG00000075292	ZNF638	0.03507	0.390828	RI
ENSG00000080802	CNOT4	0.028018	0.423203	RI
ENSG00000092199	HNRNPC	0.037872	-0.38591	RI
ENSG00000099995	SF3A1	0.028018	-0.44176	RI
ENSG00000119314	PTBP3	0.033171	0.40685	RI
ENSG00000122566	HNRNPA2B1	0.041666	0.379815	RI
ENSG00000125970	RALY	0.026872	-0.46183	RI
ENSG00000127054	CPSF3L	0.028018	-0.4304	RI
ENSG00000136709	WDR33	0.041643	0.380642	RI
ENSG00000147274	RBMX	0.037199	0.388232	RI
ENSG00000149016	TUT1	0.041643	-0.44476	RI
ENSG00000153914	SREK1	0.026238	0.479294	RI
ENSG00000174720	LARP7	0.028018	0.441012	RI
ENSG00000197111	PCBP2	0.03349	-0.39598	RI

S5 Table. **Correlation of Changes in Gene Expression of Splicing Factors and Proportion of RNA Processing Events.**

Log-fold changes in gene expression of 14 splicing factors is correlated to average $\Delta\Psi$ across three individuals for RI events.
