# Supplementary material for: Environmental perturbations lead to extensive directional shifts in RNA processing
Source: PLoS Genet. 2017 Oct 12;13(10):e1006995. doi: 10.1371/journal.pgen.1006995 (PMC5667937; doi:10.1371/journal.pgen.1006995)
Supplement: S6 Table — Log-fold changes in gene expression of 328 transcription factors is correlated to average ΔΨ across three individuals for AFE events. (PDF) [file pgen.1006995.s011.pdf]

Transcription Factor	Gene Symbol	BH FDR	Spearman's ρ	Splice Type
ENSG00000005513	SOX8	0.030831	0.501961	AFE
ENSG00000005801	ZNF195	0.007605	-0.40907	AFE
ENSG00000005889	ZFX	0.003345	-0.44432	AFE
ENSG00000007080	CCDC124	0.002938	0.448996	AFE
ENSG00000009307	CSDE1	0.008942	-0.40056	AFE
ENSG00000010818	HIVEP2	0.038052	-0.34782	AFE
ENSG00000012504	NR1H4	0.042849	0.809091	AFE
ENSG00000012817	KDM5D	0.045894	0.38035	AFE
ENSG00000018869	ZNF582	0.045494	-0.32619	AFE
ENSG00000029153	ARNTL2	0.013796	-0.477	AFE
ENSG00000029363	BCLAF1	0.008431	-0.41378	AFE
ENSG00000049618	ARID1B	0.002465	-0.51535	AFE
ENSG00000050344	NFE2L3	0.015417	-0.37475	AFE
ENSG00000055609	KMT2C	0.023405	-0.35314	AFE
ENSG00000059728	MXD1	0.007176	-0.41184	AFE
ENSG00000064933	PMS1	0.015919	-0.39857	AFE
ENSG00000064961	HMG20B	0.00087	0.529545	AFE
ENSG00000066827	ZFAT	0.011722	-0.4347	AFE
ENSG00000067066	SP100	0.029784	-0.34067	AFE
ENSG00000067646	ZFY	0.038455	-0.388	AFE
ENSG00000068305	MEF2A	0.028748	-0.34245	AFE
ENSG00000070444	MNT	0.024099	0.352026	AFE
ENSG00000071564	TCF3	0.001062	0.529317	AFE
ENSG00000071655	MBD3	0.000249	0.556077	AFE
ENSG00000072310	SREBF1	0.003345	0.519896	AFE
ENSG00000073614	KDM5A	0.049141	-0.31083	AFE
ENSG00000074657	ZNF532	0.032216	-0.3384	AFE
ENSG00000075407	ZNF37A	0.028599	0.420698	AFE
ENSG00000077463	SIRT6	0.015729	0.378564	AFE
ENSG00000077809	GTF2I	0.001436	-0.52524	AFE
ENSG00000078900	TP73	0.00713	0.717391	AFE
ENSG00000079432	CIC	0.001905	0.520348	AFE
ENSG00000080298	RFX3	0.020701	-0.42354	AFE
ENSG00000082258	CCNT2	0.002938	-0.44965	AFE
ENSG00000083838	ZNF446	0.015997	0.443737	AFE
ENSG00000085274	MYNN	0.026408	-0.34757	AFE
ENSG00000085276	MECOM	0.010629	0.40234	AFE
ENSG00000085644	ZNF213	0.01477	0.444893	AFE
ENSG00000086065	CHMP5	0.01942	-0.3628	AFE
ENSG00000087510	TFAP2C	0.035417	0.797203	AFE
ENSG00000089902	RCOR1	0.011211	-0.38909	AFE
ENSG00000090612	ZNF268	0.028292	-0.34611	AFE
ENSG00000092203	TOX4	0.036846	0.336626	AFE
ENSG00000095794	CREM	0.025756	-0.38082	AFE
ENSG00000095951	HIVEP1	0.038052	-0.34524	AFE
ENSG00000096401	CDC5L	0.003245	-0.44549	AFE
ENSG00000099326	MZF1	0.011668	0.419113	AFE
ENSG00000099364	FBXL19	0.001586	0.534916	AFE
ENSG00000100219	XBP1	0.033326	0.33436	AFE
ENSG00000100281	HMGXB4	0.046967	0.405822	AFE
ENSG00000100644	HIF1A	0.043907	-0.31786	AFE

Transcription Factor	Gene Symbol	BH FDR	Spearman's ρ	Splice Type
ENSG00000100811	YY1	0.001279	0.531399	AFE
ENSG00000100968	NFATC4	0.002465	0.552615	AFE
ENSG00000101126	ADNP	0.010482	-0.39345	AFE
ENSG00000101412	E2F1	0.022173	0.38517	AFE
ENSG00000102034	ELF4	0.024559	-0.36986	AFE
ENSG00000103199	ZNF500	0.020424	0.392277	AFE
ENSG00000103495	MAZ	0.002932	0.451107	AFE
ENSG00000104064	GABPB1	0.037314	-0.34949	AFE
ENSG00000104447	TRPS1	0.048605	-0.38015	AFE
ENSG00000105663	KMT2B	0.033912	0.36624	AFE
ENSG00000105698	USF2	0.008328	0.451146	AFE
ENSG00000105722	ERF	0.001351	0.486706	AFE
ENSG00000105732	ZNF574	0.001585	0.511275	AFE
ENSG00000105750	ZNF85	0.041996	-0.37022	AFE
ENSG00000105866	SP4	0.042677	-0.363	AFE
ENSG00000105967	TFEC	0.023025	-0.47418	AFE
ENSG00000108264	TADA2A	0.002091	-0.49579	AFE
ENSG00000108509	CAMTA2	0.027316	0.370037	AFE
ENSG00000109381	ELF2	0.038149	-0.33796	AFE
ENSG00000109685	WHSC1	0.03288	0.3582	AFE
ENSG00000110693	SOX6	0.028338	0.343604	AFE
ENSG00000112242	E2F3	0.046827	-0.31401	AFE
ENSG00000113658	SMAD5	0.031282	-0.35604	AFE
ENSG00000114853	ZBTB47	0.008976	0.423476	AFE
ENSG00000115568	ZNF142	0.001062	0.582849	AFE
ENSG00000115816	CEBPZ	0.037385	-0.32671	AFE
ENSG00000115966	ATF2	0.034645	-0.33134	AFE
ENSG00000116044	NFE2L2	0.012098	-0.3854	AFE
ENSG00000116731	PRDM2	0.025096	-0.39709	AFE
ENSG00000116809	ZBTB17	0.020715	0.394011	AFE
ENSG00000117010	ZNF684	0.048712	-0.36344	AFE
ENSG00000117625	RCOR3	0.020481	-0.39479	AFE
ENSG00000117713	ARID1A	0.036708	-0.37914	AFE
ENSG00000118217	ATF6	0.009424	-0.39774	AFE
ENSG00000118260	CREB1	0.027948	-0.37966	AFE
ENSG00000118412	CASP8AP2	0.014896	-0.37656	AFE
ENSG00000118620	ZNF430	0.019058	-0.40625	AFE
ENSG00000119574	ZBTB45	0.001614	0.48575	AFE
ENSG00000119725	ZNF410	0.004994	-0.42625	AFE
ENSG00000120690	ELF1	0.012098	-0.38553	AFE
ENSG00000120784	ZFP30	0.04937	0.457595	AFE
ENSG00000121413	ZSCAN18	0.031219	0.386984	AFE
ENSG00000122034	GTF3A	0.044177	-0.31736	AFE
ENSG00000123636	BAZ2B	0.003556	-0.48811	AFE
ENSG00000123933	MXD4	0.001108	0.500937	AFE
ENSG00000124459	ZNF45	0.002938	-0.46008	AFE
ENSG00000125520	SLC2A4RG	0.002938	0.460202	AFE
ENSG00000125817	CENPB	0.008958	0.399904	AFE
ENSG00000125846	ZNF133	0.015074	-0.37571	AFE
ENSG00000125850	OVOL2	0.020629	-0.35873	AFE

Transcription Factor	Gene Symbol	BH FDR	Spearman's ρ	Splice Type
ENSG00000125901	MRPS26	0.013655	0.387351	AFE
ENSG00000126012	KDM5C	0.002938	0.461902	AFE
ENSG00000126456	IRF3	0.002276	0.466144	AFE
ENSG00000126561	STAT5A	0.011964	0.462959	AFE
ENSG00000126603	GLIS2	0.005944	0.429916	AFE
ENSG00000126767	ELK1	0.046827	0.31807	AFE
ENSG00000127081	ZNF484	0.037864	-0.34586	AFE
ENSG00000127528	KLF2	0.028055	0.38489	AFE
ENSG00000129071	MBD4	0.004486	-0.43106	AFE
ENSG00000129535	NRL	0.003556	0.537348	AFE
ENSG00000129911	KLF16	0.003488	0.440371	AFE
ENSG00000130522	JUND	0.007744	0.407972	AFE
ENSG00000130726	TRIM28	0.033792	0.332969	AFE
ENSG00000130816	DNMT1	0.049141	0.339824	AFE
ENSG00000130856	ZNF236	0.025606	-0.41286	AFE
ENSG00000131196	NFATC1	0.04893	0.515599	AFE
ENSG00000131408	NR1H2	0.001585	0.480712	AFE
ENSG00000132005	RFX1	0.020458	0.59768	AFE
ENSG00000132010	ZNF20	0.04937	-0.33436	AFE
ENSG00000132749	MTL5	0.037385	0.854545	AFE
ENSG00000133250	ZNF414	0.013137	0.435869	AFE
ENSG00000133794	ARNTL	0.027553	-0.35239	AFE
ENSG00000134852	CLOCK	0.034472	-0.33174	AFE
ENSG00000135164	DMTF1	0.012622	-0.38856	AFE
ENSG00000135457	TFCP2	0.015161	-0.40642	AFE
ENSG00000135747	ZNF670	0.020458	-0.40101	AFE
ENSG00000136169	SETDB2	0.046392	-0.34139	AFE
ENSG00000136997	MYC	0.003345	0.442837	AFE
ENSG00000137309	HMGA1	0.047689	0.312937	AFE
ENSG00000137492	PRKRIR	0.045689	-0.31535	AFE
ENSG00000138336	TET1	0.038052	-0.36374	AFE
ENSG00000139154	AEBP2	0.012984	-0.38249	AFE
ENSG00000139624	CERS5	0.021556	-0.39228	AFE
ENSG00000140262	TCF12	0.035417	-0.3301	AFE
ENSG00000141510	TP53	0.020028	0.360803	AFE
ENSG00000141644	MBD1	0.007176	0.436023	AFE
ENSG00000141646	SMAD4	0.007176	-0.45472	AFE
ENSG00000142409	ZNF787	0.000582	0.523847	AFE
ENSG00000143178	TBX19	0.048029	-0.36123	AFE
ENSG00000143373	ZNF687	0.004013	0.483531	AFE
ENSG00000143799	PARP1	0.019722	0.36883	AFE
ENSG00000144366	GULP1	0.022509	0.572875	AFE
ENSG00000144485	HES6	0.005076	0.603841	AFE
ENSG00000146587	RBAK	0.00713	-0.4124	AFE
ENSG00000147124	ZNF41	0.020285	-0.36673	AFE
ENSG00000148200	NR6A1	0.019666	-0.46485	AFE
ENSG00000149050	ZNF214	0.038835	-0.61299	AFE
ENSG00000149922	TBX6	0.013655	0.525516	AFE
ENSG00000151612	ZNF827	0.027955	0.407987	AFE
ENSG00000151702	FLI1	0.020772	0.423312	AFE
ENSG00000152443	ZNF776	0.033792	-0.3537	AFE

Transcription Factor	Gene Symbol	BH FDR	Spearman's ρ	Splice Type
ENSG00000152926	ZNF117	0.044177	-0.33692	AFE
ENSG00000154655	L3MBTL4	0.013827	-0.48647	AFE
ENSG00000154832	CXXC1	0.007605	0.451504	AFE
ENSG00000156469	MTERFD1	0.048712	-0.34076	AFE
ENSG00000156853	ZNF689	0.019386	0.399277	AFE
ENSG00000158805	ZNF276	0.019976	0.426639	AFE
ENSG00000159588	CCDC17	0.033792	0.463227	AFE
ENSG00000159905	ZNF221	0.045894	-0.38753	AFE
ENSG00000159917	ZNF235	0.011509	-0.4293	AFE
ENSG00000160113	NR2F6	0.000353	0.58217	AFE
ENSG00000160199	PKNOX1	0.027948	-0.3451	AFE
ENSG00000160336	ZNF761	0.003952	-0.43562	AFE
ENSG00000160352	ZNF714	0.036936	-0.37815	AFE
ENSG00000160908	ZNF394	0.002465	0.497584	AFE
ENSG00000161298	ZNF382	0.033326	0.454339	AFE
ENSG00000162086	ZNF75A	0.028642	-0.38295	AFE
ENSG00000162613	FUBP1	0.028835	-0.36831	AFE
ENSG00000162714	ZNF496	0.028918	0.391245	AFE
ENSG00000162924	REL	0.004263	-0.47423	AFE
ENSG00000163795	ZNF513	0.001279	0.534307	AFE
ENSG00000163939	PBRM1	0.010997	-0.39117	AFE
ENSG00000165156	ZHX1	0.042849	-0.35141	AFE
ENSG00000165259	HDX	0.043361	-0.39527	AFE
ENSG00000165462	PHOX2A	0.036729	0.721429	AFE
ENSG00000165655	ZNF503	0.046491	0.354646	AFE
ENSG00000166188	ZNF319	0.028437	0.377692	AFE
ENSG00000166478	ZNF143	0.028055	-0.34891	AFE
ENSG00000166704	ZNF606	0.007604	0.550024	AFE
ENSG00000166888	STAT6	0.02597	0.348397	AFE
ENSG00000167232	ZNF91	0.007145	-0.41727	AFE
ENSG00000167380	ZNF226	0.036154	-0.329	AFE
ENSG00000167384	ZNF180	0.002465	-0.48598	AFE
ENSG00000167394	ZNF668	0.001614	0.491488	AFE
ENSG00000167491	GATAD2A	0.008942	0.400448	AFE
ENSG00000167548	KMT2D	0.009982	0.450831	AFE
ENSG00000167625	ZNF526	0.011895	0.388664	AFE
ENSG00000167685	ZNF444	0.00387	0.481224	AFE
ENSG00000167967	E4F1	0.000206	0.566237	AFE
ENSG00000168036	CTNNB1	0.036708	-0.32837	AFE
ENSG00000168286	THAP11	0.043381	0.33564	AFE
ENSG00000168813	ZNF507	0.048712	-0.31175	AFE
ENSG00000169155	ZBTB43	0.019644	-0.37402	AFE
ENSG00000169375	SIN3A	0.028642	-0.36119	AFE
ENSG00000169554	ZEB2	0.044629	-0.4052	AFE
ENSG00000169951	ZNF764	0.032907	0.415242	AFE
ENSG00000169957	ZNF768	0.001092	0.534257	AFE
ENSG00000170265	ZNF282	0.001278	0.544019	AFE
ENSG00000170653	ATF7	0.021904	-0.35606	AFE
ENSG00000170949	ZNF160	0.024244	-0.35157	AFE
ENSG00000171163	ZNF692	0.000742	0.601395	AFE
ENSG00000171169	NAIF1	0.007605	0.480789	AFE
ENSG00000171223	JUNB	0.008431	0.413755	AFE

Transcription Factor	Gene Symbol	BH FDR	Spearman's ρ	Splice Type
ENSG00000171425	ZNF581	0.000106	0.569626	AFE
ENSG00000171466	ZNF562	0.005361	-0.44815	AFE
ENSG00000171574	ZNF584	0.028642	0.356417	AFE
ENSG00000171735	CAMTA1	0.001959	-0.52587	AFE
ENSG00000172262	ZNF131	0.031168	-0.34473	AFE
ENSG00000172845	SP3	0.001339	-0.4873	AFE
ENSG00000173041	ZNF680	0.00957	-0.46382	AFE
ENSG00000173120	KDM2A	0.013717	-0.39467	AFE
ENSG00000173153	ESRRA	0.000467	0.554279	AFE
ENSG00000173451	THAP2	0.033424	0.334221	AFE
ENSG00000173917	HOXB2	0.007604	0.477639	AFE
ENSG00000174197	MGA	0.003245	-0.51799	AFE
ENSG00000174282	ZBTB4	0.020028	0.360866	AFE
ENSG00000174586	ZNF497	0.027463	0.399133	AFE
ENSG00000174652	ZNF266	0.038052	0.388961	AFE
ENSG00000174796	THAP6	0.011665	0.449003	AFE
ENSG00000175105	ZNF654	0.027553	-0.34574	AFE
ENSG00000175213	ZNF408	0.019817	0.394703	AFE
ENSG00000175387	SMAD2	0.031158	-0.33836	AFE
ENSG00000175467	SART1	9.68E-05	0.584687	AFE
ENSG00000175787	ZNF169	0.011211	-0.43706	AFE
ENSG00000176371	ZSCAN2	0.018119	0.497082	AFE
ENSG00000176946	THAP4	0.020028	0.360752	AFE
ENSG00000177030	DEAF1	0.005978	0.443874	AFE
ENSG00000177045	SIX5	0.013796	0.43331	AFE
ENSG00000177426	TGIF1	0.044484	0.427941	AFE
ENSG00000177732	SOX12	0.000742	0.535866	AFE
ENSG00000178338	ZNF354B	0.04959	-0.3614	AFE
ENSG00000178951	ZBTB7A	0.000106	0.566907	AFE
ENSG00000179111	HES7	0.015074	0.584274	AFE
ENSG00000179195	ZNF664	0.04937	0.385469	AFE
ENSG00000179588	ZFPM1	0.033326	0.334423	AFE
ENSG00000179627	ZBTB42	0.017455	0.455676	AFE
ENSG00000179922	ZNF784	0.024667	0.429929	AFE
ENSG00000179965	ZNF771	0.046814	0.346097	AFE
ENSG00000181135	ZNF707	0.018867	0.472824	AFE
ENSG00000181315	ZNF322	0.033431	-0.36782	AFE
ENSG00000181666	HKR1	0.008782	-0.45726	AFE
ENSG00000181827	RFX7	0.030573	-0.33935	AFE
ENSG00000182141	ZNF708	0.040885	-0.3281	AFE
ENSG00000182158	CREB3L2	0.01974	0.371136	AFE
ENSG00000182979	MTA1	0.000992	0.516757	AFE
ENSG00000184436	THAP7	0.00484	0.472122	AFE
ENSG00000184517	ZFP1	0.002276	-0.52085	AFE
ENSG00000184635	ZNF93	0.04893	-0.35667	AFE
ENSG00000184677	ZBTB40	0.041162	-0.32996	AFE
ENSG00000185024	BRF1	2.72E-05	0.673735	AFE
ENSG00000185122	HSF1	0.000306	0.538807	AFE
ENSG00000185129	PURA	0.018421	0.519353	AFE
ENSG00000185252	ZNF74	0.014909	0.440241	AFE

Transcription Factor	Gene Symbol	BH FDR	Spearman's ρ	Splice Type
ENSG00000185513	L3MBTL1	0.010482	0.636563	AFE
ENSG00000185668	POU3F1	0.004044	0.623681	AFE
ENSG00000185730	ZNF696	0.009369	0.453965	AFE
ENSG00000185947	ZNF267	0.011479	-0.40328	AFE
ENSG00000186017	ZNF566	0.047551	-0.3531	AFE
ENSG00000187098	MITF	0.025567	-0.44955	AFE
ENSG00000188290	HES4	0.006995	0.486093	AFE
ENSG00000188994	ZNF292	0.012281	-0.44974	AFE
ENSG00000189042	ZNF567	0.033877	-0.39072	AFE
ENSG00000189079	ARID2	0.042296	-0.31998	AFE
ENSG00000189144	ZNF573	0.016848	-0.42669	AFE
ENSG00000196150	ZNF250	0.047422	0.317441	AFE
ENSG00000196233	LCOR	0.044066	-0.33264	AFE
ENSG00000196247	ZNF107	0.012259	-0.39209	AFE
ENSG00000196268	ZNF493	0.025606	-0.38993	AFE
ENSG00000196378	ZNF34	0.029598	0.458571	AFE
ENSG00000196417	ZNF765	0.039728	0.640351	AFE
ENSG00000196418	ZNF124	0.04225	-0.34481	AFE
ENSG00000196453	ZNF777	0.011211	0.396415	AFE
ENSG00000196456	ZNF775	0.016112	0.372399	AFE
ENSG00000196498	NCOR2	0.01005	0.440091	AFE
ENSG00000196591	HDAC2	0.020028	-0.36074	AFE
ENSG00000196646	ZNF136	0.037508	-0.36802	AFE
ENSG00000196705	ZNF431	0.02772	-0.35915	AFE
ENSG00000196757	ZNF700	0.047422	-0.31347	AFE
ENSG00000196826	ZNF709	0.046827	-0.31804	AFE
ENSG00000196843	ARID5A	0.034828	0.358989	AFE
ENSG00000197008	ZNF138	0.031158	-0.37807	AFE
ENSG00000197020	ZNF100	0.006398	-0.47779	AFE
ENSG00000197050	ZNF420	0.038052	-0.36109	AFE
ENSG00000197054	ZNF763	0.048148	-0.31232	AFE
ENSG00000197362	ZNF786	0.026047	0.369447	AFE
ENSG00000197372	ZNF675	0.002298	-0.50532	AFE
ENSG00000197483	ZNF628	0.040307	0.382229	AFE
ENSG00000197497	ZNF665	0.036863	0.488196	AFE
ENSG00000197608	ZNF841	0.03285	-0.33522	AFE
ENSG00000197779	ZNF81	0.008616	-0.44462	AFE
ENSG00000197782	ZNF780A	0.004557	-0.43504	AFE
ENSG00000198026	ZNF335	0.016382	0.371754	AFE
ENSG00000198160	MIER1	0.00812	-0.40613	AFE
ENSG00000198169	ZNF251	0.042695	0.381947	AFE
ENSG00000198176	TFDP1	0.00433	0.480609	AFE
ENSG00000198182	ZNF607	0.002817	-0.54309	AFE
ENSG00000198466	ZNF587	0.033675	-0.33383	AFE
ENSG00000198517	MAFK	0.006398	0.432469	AFE
ENSG00000198521	ZNF43	0.010482	-0.41954	AFE
ENSG00000198554	WDHD1	0.049141	-0.3168	AFE
ENSG00000198815	FOXJ3	0.037839	-0.38613	AFE
ENSG00000198816	ZNF358	0.028748	0.391835	AFE
ENSG00000198911	SREBF2	0.03358	0.349606	AFE

Transcription Factor	Gene Symbol	BH FDR	Spearman's ρ	Splice Type
ENSG00000204231	RXRB	0.044904	0.336019	AFE
ENSG00000204304	PBX2	0.033729	0.356597	AFE
ENSG00000204514	ZNF814	0.017669	-0.37064	AFE
ENSG00000204519	ZNF551	0.018119	0.425798	AFE
ENSG00000204859	ZBTB48	0.001331	0.540091	AFE
ENSG00000204946	ZNF783	0.037629	0.380009	AFE
ENSG00000205903	ZNF316	0.001279	0.497756	AFE
ENSG00000213015	ZNF580	0.000249	0.558377	AFE
ENSG00000213297	ZNF625-ZNF20	0.049141	-0.32323	AFE
ENSG00000213347	MXD3	0.029827	0.340544	AFE
ENSG00000214022	REPIN1	0.002427	0.486217	AFE
ENSG00000215421	ZNF407	0.0298	0.613636	AFE
ENSG00000218891	ZNF579	0.006529	0.44006	AFE
ENSG00000225614	ZNF469	0.022173	0.403079	AFE
ENSG00000229809	ZNF688	0.011085	0.539212	AFE
ENSG00000234444	ZNF736	0.013363	-0.38812	AFE
ENSG00000236609	ZNF853	0.018577	0.578889	AFE
ENSG00000242802	AP5Z1	0.014896	0.399207	AFE
ENSG00000243943	ZNF512	0.001139	-0.49642	AFE
ENSG00000244405	ETV5	0.035739	-0.3975	AFE
ENSG00000249459	ZNF286B	0.018236	0.526675	AFE
ENSG00000249709	ZNF564	0.042849	-0.33646	AFE
ENSG00000250571	GLI4	0.00386	0.543777	AFE
ENSG00000251369	ZNF550	0.036936	0.431869	AFE

S6 Table. **Correlation of Changes in Gene Expression of Transcription Factors and Proportion of RNA Processing Events.**

Log-fold changes in gene expression of 328 transcription factors is correlated to average $\Delta\Psi$ across three individuals for AFE events.
